# Techniques for Measurement of Serotonin: Implications in Neuropsychiatric Disorders and Advances in Absolute Value Recording Methods

**DOI:** 10.1021/acschemneuro.3c00618

**Published:** 2023-11-29

**Authors:** Juan M. Rojas Cabrera, Tyler S. Oesterle, Aaron E. Rusheen, Abhinav Goyal, Kristen M. Scheitler, Ian Mandybur, Charles D. Blaha, Kevin E. Bennet, Michael L. Heien, Dong Pyo Jang, Kendall H. Lee, Yoonbae Oh, Hojin Shin

**Affiliations:** † Medical Scientist Training Program, Mayo Clinic, Rochester, Minnesota 55902, United States; ‡ Department of Neurologic Surgery, Mayo Clinic, Rochester, Minnesota 55902, United States; § Department of Biomedical Engineering, Mayo Clinic, Rochester, Minnesota 55902, United States; ∥ Department of Psychiatry and Psychology, Mayo Clinic, Rochester, Minnesota 55902, United States; ⊥ Robert D. and Patricia K. Kern Center for the Science of Health Care Delivery, Mayo Clinic, Rochester, Minnesota 55902, United States; # Department of Biomedical Engineering, Hanyang University, Seoul 04763, South Korea; ¶ Division of Engineering, Mayo Clinic, Rochester, Minnesota 55902, United States; ∇ Department of Chemistry and Biochemistry, University of Arizona, Tucson, Arizona 85721, United States

**Keywords:** Serotonin, electrochemistry, N-MCSWV, FSCV, FSCAV, addiction, schizophrenia, depression, MDD, opioids

## Abstract

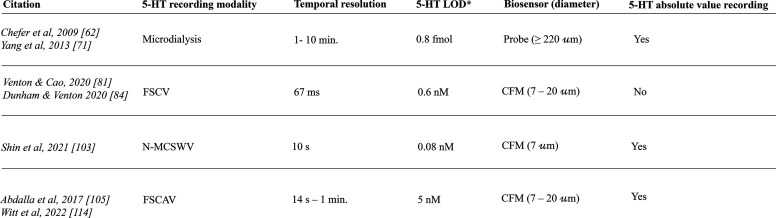

Serotonin (5-HT) is a monoamine neurotransmitter in the peripheral, enteric, and central nervous systems (CNS). Within the CNS, serotonin is principally involved in mood regulation and reward-seeking behaviors. It is a critical regulator in CNS pathologies such as major depressive disorder, addiction, and schizophrenia. Consequently, *in vivo* serotonin measurements within the CNS have emerged as one of many promising approaches to investigating the pathogenesis, progression, and treatment of these and other neuropsychiatric conditions. These techniques vary in methods, ranging from analyte sampling with microdialysis to voltammetry. Provided this diversity in approach, inherent differences between techniques are inevitable. These include biosensor size, temporal/spatial resolution, and absolute value measurement capabilities, all of which must be considered to fit the prospective researcher’s needs. In this review, we summarize currently available methods for the measurement of serotonin, including novel voltammetric absolute value measurement techniques. We also detail serotonin’s role in various neuropsychiatric conditions, highlighting the role of phasic and tonic serotonergic neuronal firing within each where relevant. Lastly, we briefly review the present clinical application of these techniques and discuss the potential of a closed-loop monitoring and neuromodulation system utilizing deep brain stimulation (DBS).

## Introduction

### Serotonin

Serotonin (5-hydroxytryptamine, 5-HT) is a monoamine neurotransmitter derived from the α-amino acid l-tryptophan. As this neurochemical is hydrophilic, it is unable to cross the lipophilic blood–brain barrier (BBB) and thus forms two functionally distinct pools in the central nervous system (CNS) and peripheral tissues.^1^ Tryptophan hydroxylase is the enzyme responsible for catalyzing the conversion of l-tryptophan into 5-hydroxy-l-tryptophan, the rate-limiting step in synthesizing serotonin. In the gastrointestinal system, serotonin is synthesized by tryptophan hydroxylase 1 (Tph1), which is predominantly expressed by the neuroendocrine enterochromaffin cells that line the lumen of the small intestine and colon.^1^ Enteric serotonin regulates gut motility via smooth muscle contractions, mediates the local immune system response, and is involved in glucose metabolism.^2^ Of note, serotonin concentrations in the GI system account for more than 95% of the total amount in the human body.^1^ Serotonin also plays a minor role in the peripheral nervous system (PNS), influencing cardiovascular functioning, immune functioning, and pain perception.^3^ In the CNS, serotonin is synthesized by tryptophan hydroxylase 2 (Tph2). Serotonergic neurons express Tph2 in the raphe nuclei, a bilateral reticular formation structure in the midbrain with extensive serotonergic projections to regions such as the forebrain and spinal cord.^4^ This vast distribution may explain serotonin’s numerous involvements within normal-state CNS functions like mood and appetite regulation, thermoregulation, sexual function, and reward-seeking behaviors.^5,6^

### Impact of Serotonin Today

Public interest in investigating neuropsychiatric conditions like depression and substance use disorders (SUD) has increased dramatically in the past decade, with factors like an uptick in mental illness prevalence among children and adults as a result of the COVID-19 pandemic and social media use likely contributing to this growth.^7−10^ Regarding SUD specifically, the neurotransmitter dopamine and its role in mediating addictive behaviors have been extensively researched.^11−17^ Serotonergic regulation on dopaminergic neurotransmission has also been demonstrated in detail.^18^ Unfortunately, the literature investigating serotonin modulation within addiction animal models has been sparse. However, recent *in vivo* studies have rekindled the interest in better understanding serotonin’s signaling dynamics in both normal and SUD states and other neuropsychiatric conditions.^19−21^

The interest (or urgency) in SUD research is further amplified by events such as the opioid epidemic, an ongoing, worldwide public health emergency. In the United States alone over 100 000 deaths have been attributed to opioid drug overdoses, a number that continues to rise each year and is on par with or outpaces other causes of death like diabetes, liver, and renal disease.^22^ Recent data compiled by the CDC has also shown an alarming rise in deaths stemming from potent synthetic opioids like fentanyl which now overshadows the deaths attributed to prescription opioids, cocaine, and methamphetamines.^22^ The impact of the opioid epidemic has also had detrimental effects on the economy. Recent estimates from the CDC have found that epidemic-related deaths, healthcare access, criminal justice, and diminished quality of life cost the U.S. economy over one trillion dollars in the 2017 fiscal year.^23^

Such substantial impacts on daily living paired with the rapidly evolving literature showcasing the key role of serotonin in the disease have inevitably led many within the fields of public health and biomedical research to begin exploring methods of monitoring this neurotransmitter specifically within the CNS. This approach would aid in addressing major health issues like the opioid epidemic through the conceptualization of novel treatments, elucidation of the current treatment modalities’ mechanism of action, and a better understanding of the biological pathways associated with the disease and associated comorbidities. However, before reviewing available CNS monitoring techniques, it is crucial to provide context by highlighting serotonin’s role within various neuropsychiatric disease states such as major depressive disorder, schizophrenia, and substance use disorders.

## Serotonin in Neuropsychiatric Diseases

### Major Depressive Disorder

Major depressive disorder (MDD) is a neuropsychiatric disease characterized by dysphoria, anhedonia, impaired cognition, reduced energy levels, and decreased quality of sleep and eating habits.^24^ A clinical diagnosis of MDD is made when an individual experiences at least five of the nine symptoms detailed in the DSM-5.^25^ Of these, at least one must include either anhedonia or depressed mood symptoms, and all symptoms must persist for at least 2 weeks. The lifetime prevalence of MDD ranges from 5 to 17% and is twice as prevalent in women. According to the WHO, MDD has been identified as one of the leading causes of diminished quality of life worldwide irrespective of a person’s wealth or race.^24^ Recent studies have also demonstrated that MDD increases the incidence of cardiovascular disease and diabetes while concomitantly worsening these and other conditions like autoimmune disorders and addiction.^26^ Thus, MDD’s prevalence and detriment to the general population make it a prime target for treatment refinement and the development of novel approaches to address the disease.

Although the exact cause of MDD is not known, multiple factors including an individual’s environment, lifestyle, and genetics play significant roles in the onset of the disease.^24^ Dysregulation in the serotonergic system has been hypothesized as a causal factor of MDD, often termed the serotonin hypothesis and first proposed by Alec Coppen in his 1967 paper.^27^ This hypothesis postulates that abnormalities within the serotonergic system, like decreased synaptic serotonin concentrations, can lead to depression and other associated symptoms of MDD.^27−29^ Nevertheless, the argument that decreased serotonin levels lead to the pathogenesis of MDD has been inconclusive at best. On one hand, multiple studies have shown that increasing synaptic concentrations of serotonin via pharmacologic manipulation led to decreases in patients’ depression severity.^30−33^ In fact, the latter is the basis for using SSRIs, which inhibit the presynaptic reuptake of serotonin by the serotonin transporter (SERT) as pharmacological treatment for MDD.^34^ In their work conducted by Lindseth and colleagues they found that patients on low tryptophan diets had lower affective scores (an indicator of mood) than those on a high tryptophan diet.^35^ The role of tryptophan concentrations in MDD has also been highlighted in studies showing patients with more severe MDD had lower serum levels of tryptophan compared to those with less severe MDD.^36^ However, some studies show that a high tryptophan diet does not itself cause decreased depressiveness but rather has neuroprotective effects that prevent the onset of depression, while others have shown that tryptophan restrictions within healthy individuals with no medical history of MDD had no or variable effects on their mood.^37−39^

Early studies investigating the role of tonic serotonin in MDD date back to the late 1990s. Haddjeri and colleagues demonstrated that chronic use of SSRIs and tricyclic antidepressants (TCAs) tonically activated postsynaptic 5-HT_1A_ receptors and caused increased firing of CA_3_ pyramidal neurons within the dorsal hippocampus, an unexpected finding contrary to 5-HT_1A_’s presumed function at the time.^40^ These results indicated that antidepressants may exert their therapeutic effects via previously unexplored mechanisms. Other studies have also shown that serotonin, both phasic and tonic, plays an important role in MDD-associated behaviors, such as aversion processing. Computational models hypothesize that phasic serotonergic responses serve as indicators for when events have worse outcomes than expected, while tonic responses serve as averages of punishment expectations by the individual.^41^ These findings paired with serotonin’s potential role in behavioral inhibition may explain behavioral inhibition to aversive experiences like social isolation.^41^

### Schizophrenia

Schizophrenia is a neuropsychiatric disorder that affects 5 in every 1000 people in the United States.^42^ It is equally prevalent among males and females, although males do tend to experience schizophrenic symptoms earlier in their lives.^43^ Symptoms associated with schizophrenia can be divided into two broad categories: negative and positive.^44^ Negative symptoms include anhedonia, apathy, and decreases in cognitive processing. Positive symptoms include delusional behavior, hallucinations, and abnormal psychomotor behaviors. An individual with suspected schizophrenia is formally diagnosed when they experience two or more of the five DSM-5 characteristic symptoms for more than one month, among additional criteria.^25^

Abnormal concentrations of dopamine and serotonin have been implicated in the pathogenesis of schizophrenia.^45,46^ Elevated levels of dopamine in the mesolimbic pathway have been linked to the onset of positive symptoms, while decreased levels of dopamine in the nigrostriatal and mesocortical pathways cause extrapyramidal (motor) and negative symptoms, respectively.^47^ Atypical antipsychotics like haloperidol alleviate positive symptoms, presumably through antagonism of D_2_ dopamine receptors and decreased dopaminergic signaling.^48^ While effective, these drugs have significant side effects, including exacerbation of extrapyramidal dysfunction and negative symptoms. The development of drugs like clozapine (termed second generation “atypical” antipsychotics) has resulted in a better reduction of schizophrenic symptoms via both dopamine and serotonin receptor antagonism.^49^ Interest in serotonin and its role in disease has increased as a result of these advances.

The pathogenesis of schizophrenia is poorly understood, although studies in brain development and clinical trials may provide valuable insight. A study by Zhang, 2003, showed that serotonin caused tonic firing in the prefrontal cortex during development, indicating that serotonin may play a role in its differentiation.^50^ This finding is significant as deficiencies in the prefrontal cortex are associated with schizophrenic symptoms. Randomized control clinical trials using deep brain stimulation (DBS) for treatment-resistant schizophrenia have also shown promising results by alleviating symptoms.^51^ Unfortunately, the specific roles of tonic and phasic-firing serotonergic neurons in schizophrenia during development and treatment implementation have not been investigated in depth.

### Substance Use Disorder

A substance use disorder (SUD) is characterized by physical and psychological dependence on substances, such as alcohol (EtOH), stimulants, nicotine, and opioids. A clinical diagnosis occurs when an individual meets at least 3 of the seven DSM-5 criteria, which include negative consequences associated with use, cravings, tolerance to the substance, withdrawal effects, and the recurrence of physical and psychological disturbances.^52^ Substance-seeking behaviors and altered cognitive states associated with SUD can worsen over time and significantly impact an individual’s health and social well-being, resulting in a diminished quality of life and potential death. SUD treatments are highly dependent on the drug of abuse but generally include pharmacological and psychotherapy.^53^

Like MDD, the pathogenesis of SUD is highly influenced by environmental, genetic, and neurobiological factors.^54^ Environmental factors that can lead to addiction include the presence of drugs of abuse in the individual’s space, exposure at a young age, the consumption of these drugs within a person’s family or another close social group, and social media influences.^55−57^ Although these environmental risk factors will not be discussed in detail here, their impact on SUD is indispensable when discussing other factors leading to addiction. As for serotonin in addiction, its exact role is not fully understood. However, recent studies heavily implicate it in addictive behaviors. In a study published by Li and colleagues, the group found that inhibiting cocaine-SERT binding resulted in increased compulsive behaviors during self-administration of the drug.^19^ These behaviors were reversed by using the SSRI citalopram. Their evidence suggested that the behavior change was due to presynaptic depression via the serotonin receptor 5-HT_1B_ in the dorsal striatum. This results in decreased glutamate transmission and modulation of compulsion. Phasic and tonic serotonins were not recorded in this study. In a separate 2022 study, Yuen and colleagues found that following cocaine administration, the stimulation-evoked release of serotonin was amplified in the nucleus accumbens.^58^ Unfortunately, further studies that elucidate the mechanisms behind this change in serotonin dynamics, tonic levels of the neurotransmitter in response to drug administration, and associated behaviors *in vivo* are currently unavailable.

## Serotonin Measurement Techniques

Despite our current understanding of serotonergic signaling in neuropsychiatric illnesses, long-term pharmacological modulation of tonic extracellular levels of serotonin in the brain, a core component mediating therapeutic responses, remains relatively unexplored.^59−61^ The reason for this, in part, is that such studies would require significant advances in *in vivo* neurotransmitter monitoring techniques. In the following section, we will discuss these various techniques and potential limitations in applying them for clinical use with a focus on novel absolute value measurements. A summary of the techniques discussed herein and important features is in Table 1.

**Table 1:** Recording Techniques Used for *in Vivo* Serotonin Measurements and Their Associated Temporal Resolution, Limit of Detection, Biosensor Type/Diameter, and Serotonin Absolute Value Measurement Capabilities^a^

citation	5-HT recording modality	temporal resolution	5-HT LOD^b^	biosensor (diameter)	5-HT absolute value recording
Chefer et al., 2009^62^	microdialysis	1–10 min	0.8 fmol	probe (≥220 μm)	yes
Yang et al., 2013^71^					
Venton and Cao, 2020^81^	FSCV	67 ms	0.6 nM	CFM (7–20 μm)	no
Dunham and Venton 2020^84^					
Shin et al., 2021^103^	N-MCSWV	10 s	0.08 nM	CFM (7 μm)	yes
Abdalla et al., 2017^105^	FSCAV	14 s to 1 min	5 nM	CFM (7–20 μm)	yes
Witt et al., 2022^114^					

a Abbreviations: LOD = level of detection, fmol = femtomole, nM = nanomolar, CFM = carbon fiber microelectrode.

b The lowest LOD found in the literature review was listed for each technique.

### Microdialysis and HPLC

Some of the earliest *in vivo* CNS measurements of serotonin were performed using microdialysis in the 1970s and 1980s.^62^ Microdialysis in its simplest form utilizes a concentric probe with a semipermeable membrane inserted into the area of interest within the brain. When a dialysate is perfused at a slow, constant rate (usually between 0.1–3.0 μL/min), the analyte of interest diffuses down a concentration gradient through the membrane where its absolute concentration can be quantified either online or offline.^63^ Due to the direct capture and subsequent identification of molecules, microdialysis is a highly appealing technique for extracellular chemical detection *in vivo* within a plethora of tissues and animal species.

High-performance liquid chromatography (HPLC) is often paired with microdialysis to analyze serotonin *in vivo*.^64,65^ HPLC has two major components: a stationary phase, composed of adsorbent materials, and a mobile phase which is a pressurized liquid injected into the column.^66^ Depending on the adsorption properties of the stationary phase and the interactions with the molecules in the mobile phase, these components will flow out of the column at different rates and thus separate from each other. The molecules can then be identified by using various types of detectors, including mass spectroscopy, fluorescence, and UV light.

For serotonin analysis, microdialysis cannulas can be inserted into tissues such as the brain, uterus, intestines, or colon of animals. Generally, a dialysate (made up of deionized H_2_O, saline, and 5% bovine serum albumin) is then perfused through the cannulas. Once extracted, the dialysate is analyzed using HPLC to identify l-5-hydroxytryptophan (5-HTP), a precursor to serotonin. Many publications have utilized this technique to monitor serotonin, including recent investigations of serotonin levels following pharmacological manipulation with selective serotonin reuptake inhibitors (SSRIs) and in zebrafish exposed to ethanol.^67,68^ HPLC-UV is a slight modification to HPLC in that instead of using an electrochemical detector it utilizes a UV-light detector to identify the molecule.^69^ Benefits of this technique include decreased noise interference during the sampling process and utilization of more inexpensive chemicals compared to traditional HPLC all while maintaining comparable sensitivity to similar methods.^70^

Advances in microdialysis have allowed for enhanced serotonin collection including decreased detection thresholds and faster sampling rates of the neurotransmitter. One such advancement is detailed by Yang and colleagues who developed a platform with a serotonin detection threshold of 0.8 fmol and a sampling rate of 6 min, all while benefiting from concurrent recording and analysis of absolute levels of serotonin and dopamine.^71^ Other notable modifications include those achieved by El-Sherbeni and colleagues who have improved the biostability of catecholamines during the collection process, allowing for sustained measurement *in vivo*.^72^ Zestos and colleagues detail additional advancements to microdialysis in its application for epilepsy studies in their review. Notable improvements include the use of segmental flow to increase temporal resolution, down-sizing of the dialysis probe to minimize tissue damage, and development of concomitant optogenetic modulation and dialysate sampling.^73^

Despite possessing absolute value measurement capabilities and a proven track record in *in vivo* applications, microdialysis has inherent limitations in its use for *in vivo* serotonin measurement. These include a low temporal resolution (≥1 min) and a large probe size (typically >200 μm diameter, >1 mm length), the latter of which can cause extensive damage to the brain tissue.^74−80^ Microdialysis also requires continuous extraction of dialysate samples from the brain, making its application in the human nervous system for chronic recordings cumbersome. HPLC shares similar limitations to those of microdialysis detailed above, as it utilizes microdialysis for the sample collection process. However, HPLC also generally requires the use of additional pieces of equipment for its traditionally offline analysis methods including a pump, column, and detector.

### Fast-Scan Cyclic Voltammetry

Fast-scan cyclic voltammetry (FSCV) is an electrochemical technique with a well-established track record in measuring real-time, subsecond changes in serotonin, dopamine, and other electroactive chemicals’ concentrations. As the name implies, FSCV is a modified version of conventional cyclic voltammetry via its higher scan rate (∼100 mV/s for conventional CV compared to >400 V/s for FSCV).^81^ This modification is vital to recording neurotransmitters that function at higher time scales. To measure neurotransmitters with FSCV, a working electrode, typically made of carbon fiber, is placed into the solution or tissue of interest. A waveform with distinct switching potentials is then applied at a speed of 10 Hz, causing molecules to be electro-oxidized in the forward-sweep (positive voltage) and electroreduced to their initial form during the backward-sweep (negative voltage). The background charging current that FSCV produces is then subtracted, leaving only the faradic current which can then be plotted on a voltage vs time graph for visualization. As most electroactive molecules can be oxidized and reduced at distinct potentials, the waveform utilized in FSCV can be modified to capture specific molecules.^81,82^

Early recordings of serotonin with FSCV utilized the “Jackson” waveform.^83^ This waveform, which is typically applied at a rate of 1000 V/s, has sequential switching potentials of +0.2, +1.0, −0.1, and back to +0.2 V which gives it its characteristic “N” shape. This waveform was developed to increase the selectivity of serotonin over its metabolites, such as 5-hydroxyindoleacetic acid (5-HIAA). 5-HIAA and other radicals produced during the redox reaction can accumulate on the surface of the carbon fiber biosensor during recording sessions, thereby decreasing its sensitivity to serotonin over time and through repeated exposure to these metabolites. Using a fast scan rate, the Jackson waveform reduced this biofouling effect by decreasing the adsorption of serotonin to the carbon fiber and, in turn, decreasing the number of serotonin byproducts produced during its redox reaction. Optimization of the Jackson waveform has resulted in modified waveforms that have reduced electrode biofouling and increased serotonin selectivity.^61,84^ Further biosensor modifications such as the electrodeposition of the polymer Nafion to the carbon fiber have also increased serotonin selectivity by mitigating biofouling and increasing its cation species selectivity.^85^

FSCV possesses some features that are better suited to quantitatively measure changes in extracellular neurotransmitter concentrations in chronic or behavioral studies, including fewer equipment needs relative to other techniques and implantation versatility.^83,86−88^ Additionally, the typical carbon fiber microelectrode used with FSCV is smaller (7 μm in diameter, 50–150 μm in length), thus providing micrometer spatial resolution of detection in tissue with less damage. FSCV also benefits from a subsecond temporal response and low limits of detection (e.g., <5 nM) for serotonin, though techniques like microdialysis have significantly lower limits.^89−92^ Like microdialysis, FSCV has also been applied to several animal-based disease models and is well-established within their respective fields.^93−98^ However, a noteworthy drawback to FSCV is that because conventional FSCV requires background subtraction of tonic redox currents, it has been limited to the measurement of neurochemical phasic changes induced by relatively brief bouts of chemical or electrical stimulation of neuronal elements in tissue. Thus, the requirement for background subtraction can only provide relative changes to the concentration of the analyte, not their absolute value.

### Multiphoton Microscopy

Three-photon excitation is a technique developed in the 1990s that enables direct visualization of serotonergic neurons, allowing for the quantification of vesicular serotonin concentrations in intact cells.^99^ This technique works through the excitation of a fluorophore such as serotonin with high-intensity irradiation using infrared light. This causes the molecule to undergo autofluorescence, and the light emitted is subsequently detected by a three-photon absorption mechanism that provides a three-dimensional resolution of the molecule. A unique feature of this technique is the ability to visualize vesicular serotonin directly and, thus, allows researchers to directly monitor changes in serotonin synthesis within the neuron in response to stimuli and treatments. An additional benefit of multiphoton microscopy is the capability to quantify other molecules like tryptophan, dopamine, and norepinephrine.^100^

An advantage of multiphoton microscopy is the lack of a probe, meaning that it is much less invasive than any technique that requires direct insertion of its biosensor into tissue. However, it too has extensive equipment requirements, including lasers ultrasensitive to environmental disturbances.^99^ Additionally, many forms of this technique require the tissue of interest to be isolated from its host and placed in a reservoir for the fluorescence measurement. Though advancements have been made to these techniques, their implementation in human subjects to investigate neuropsychiatric illnesses and other neurological disorders in the future is still a work in progress.^67,101,102^

### N-Shaped Multiple Cyclic Square Wave Voltammetry

**Figure 1:**
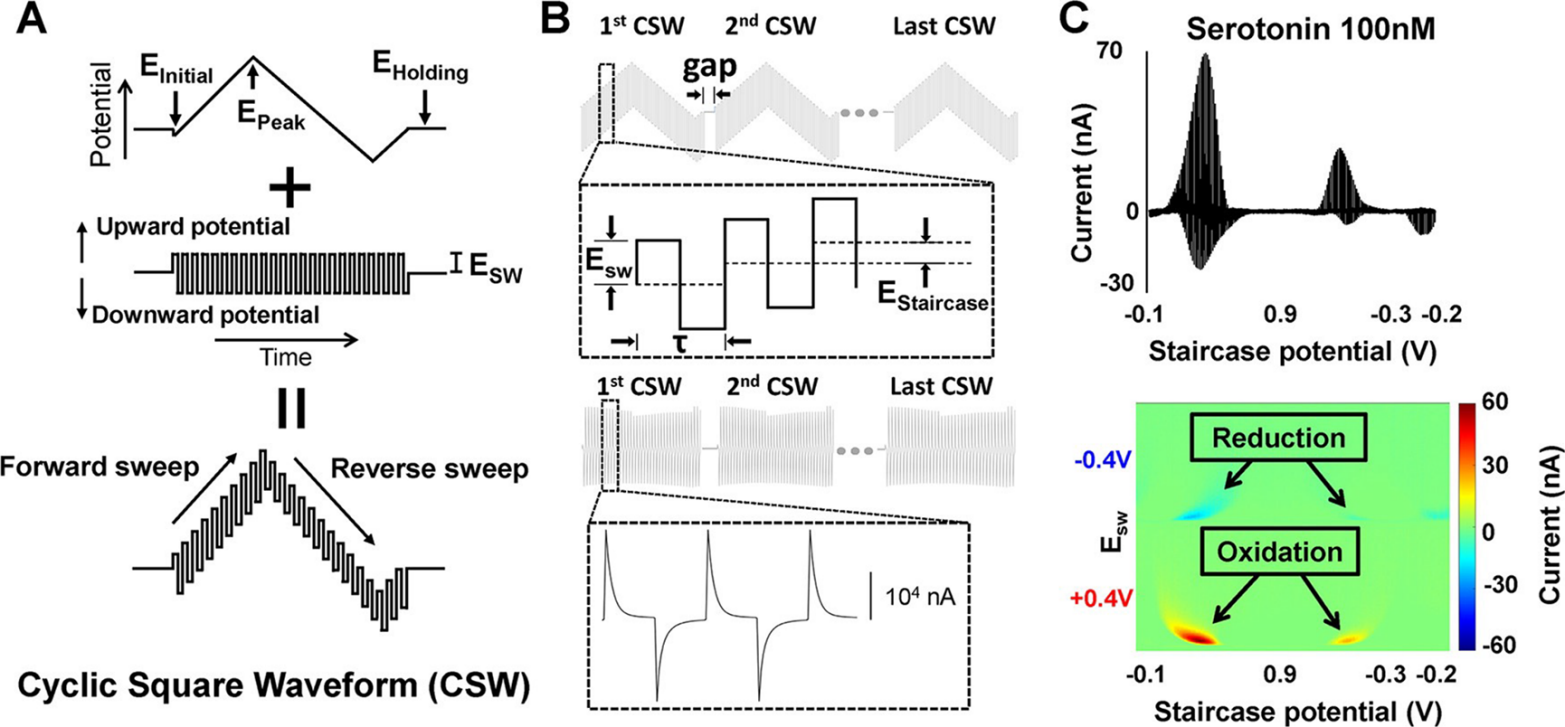
Schematic of the waveform utilized in N-MCSWV and depiction of dynamic background subtraction (A, B). A voltammogram with serotonin’s oxidation and reduction is depicted as a color plot (C). Reprinted (adapted) with permission from Hojin Shin, Abhinav Goyal, Analytical Chemistry202193 ( (51), ), 16987–1699434855368 10.1021/acs.analchem.1c02131PMC9393895. Copyright 2021 American Chemical Society.

N-shaped multiple cyclic square wave voltammetry (N-MCSWV) is a technique recently reported by our group that enables analytical quantitation of serotonin concentrations in vivo.^103^ The development of N-MCSWV is in its simplest form a combination of the N-shaped waveform used in fast cyclic square wave voltammetry for serotonin detection and the principles of modified cyclic square wave voltammetry used in M-CSWV, a tonic dopamine recording technique also developed by our group.^104^ N-MCSWV utilizes cyclic square wave voltammetric waveforms superimposed onto an N-shaped waveform with switching potentials that capture the oxidation and reduction of serotonin (Figure 1a). The amount of serotonin adsorbed to the surface of the CFM is quantified following dynamic background subtraction, which involves subtracting the second waveform from the last waveform applied, resulting in a predominantly faradic current (Figure 1b). However, as the cyclic square waves applied contain small amounts of nonfaradic current due to their capacitive charging current, N-MCSWV simulates the capacitive charging current from the cyclic square waveform and subtracts it from the redox response. The result is the quantification of the absolute value of tonic serotonin concentrations and a voltammogram-derived color plot for data visualization (Figure 1c).

N-MCSWV functions with basic voltammetry principles and, thus, shares many of the benefits associated with related electrochemical techniques. For example, N-MCSWV possesses a higher temporal resolution (10 s) relative to other tonic serotonin measurement techniques (20 seconds to minutes).^103,105−109^ The N-MCSWV waveform described above also cycles through the N-shaped waveform multiple times and performs multiple redox reactions, resulting in increased selectivity for serotonin against electroactive interferents such as 5-HIAA, histamine, norepinephrine, ascorbic acid, and pH changes (LOD = 0.08 nM). This method also exploits the adsorption characteristics of serotonin at the surface of CFMs over other electroactive catecholamines, such as dopamine, to enable selective measurements of tonic serotonin concentrations.

### Fast-Scan Controlled-Adsorption Voltammetry

**Figure 2:**
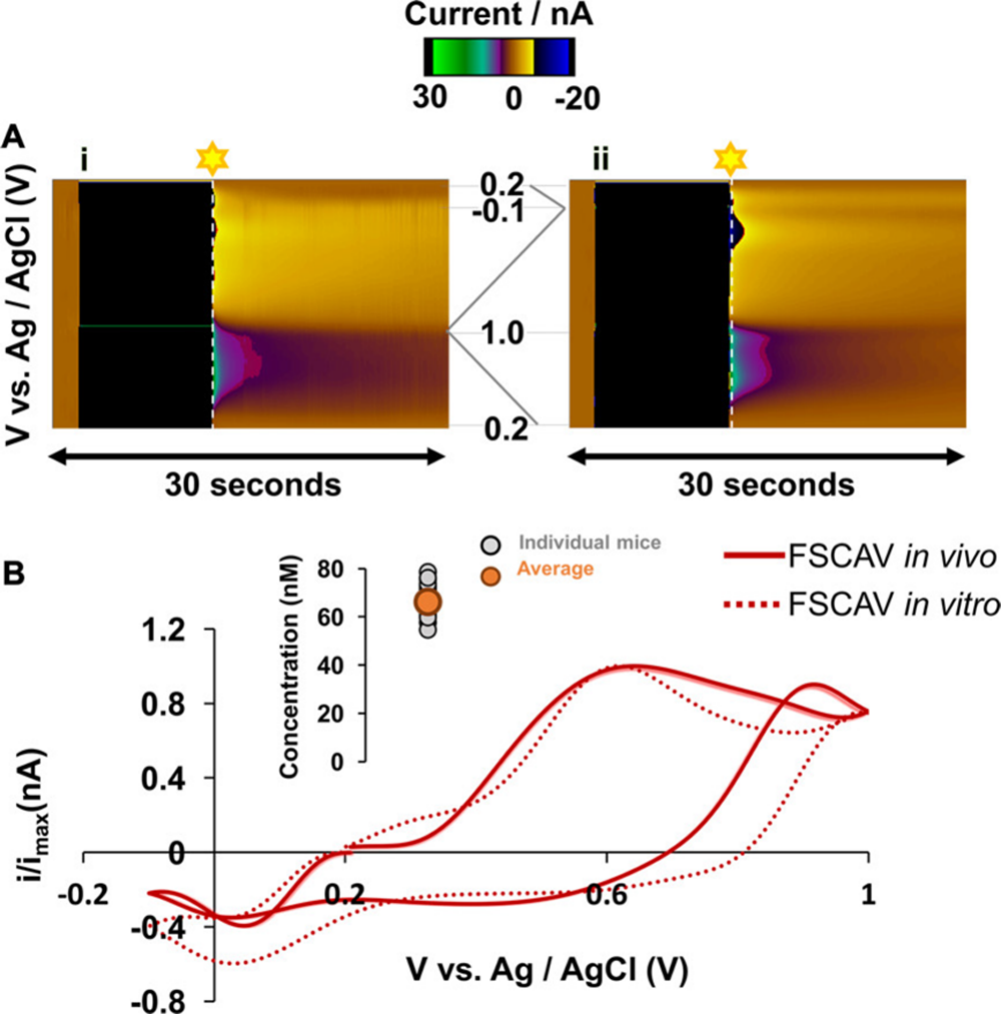
In vivo (Ai) and in vitro (Aii) FSCAV color plots. Black bars indicate the 10 s controlled adsorption period. Cyclic voltammograms captured at 100 Hz for detection of the adsorbed molecule, serotonin, are colored red (B). The voltammogram captured at 100 Hz *in vitro* and *in vivo* is indicated by the dotted and solid lines, respectively. The inset in panel B shows the serotonin concentration measurements obtained from the mouse hippocampal CA2 region (*n* = 15). Reprinted (adapted) with permission from Aya Abdalla, Christopher W. Atcherley, Pavithra Pathirathna, Analytical Chemistry201789 ( (18), ), 9703–971128795565 10.1021/acs.analchem.7b01257PMC5837047.^105^ Copyright 2017 American Chemical Society.

Fast-scan controlled-adsorption voltammetry (FSCAV) is a technique developed by modifying FSCV principles and exploiting the adsorption properties of carbon fiber microelectrodes, thus enabling the measurement of tonic neurotransmitter concentrations *in vivo*.^103−105,110−113^ To achieve this, FSCAV first applies a “Jackson” waveform for serotonin detection every 10 ms (100 Hz). At this frequency, the adsorption of neurochemicals to the carbon fiber is minimized. The system then switches to a holding potential of 0.2 V which is applied for at least 10 s, allowing the surface concentration of the analyte adsorbed onto the carbon fiber to reach equilibrium. The initial waveform is then applied once again at 100 Hz, allowing the adsorbed molecule’s redox potential peaks to be fully captured. Finally, FSCAV acquisition ends with a 30 s waiting period. As previously discussed, the background subtraction of a large capacitive current performed with FSCV inherently prevents the measurement of absolute electrochemical concentration levels. However, FSCAV solves this by subtracting the initial voltammogram with minimal adsorption of the neurochemical from the voltammogram obtained after the holding potential delay. This results in the separation of faradic and nonfaradic current and provides the absolute value measurement of the neurochemical adsorbed onto the carbon fiber. Figure 2 provides a schematic detailing the waveform application process used in FSCAV specifically for *in vivo* serotonin detection.

In the context of serotonin monitoring, FSCAV has been utilized to measure basal levels of serotonin *in vivo* within the CA2 region of the mouse hippocampus and prefrontal cortex, as well as monitoring of serotoninergic changes following pharmacologic challenge.^105,114−116^ Notable advancements of FSCAV include its use in *in vivo* serotonin analysis with an artificial neural network (AAN) developed by Hashemi and colleagues.^117^ Using this AAN, absolute value measurements of serotonin were successfully obtained with low predictive error using in vivo data obtained with electrodes of varying sensitivities. FSCAV data are also notorious for its technically difficult postanalysis process. However, within this same work, Hashemi and co-workers provide a fully automated, Web-based, open-source platform that simplifies the user experience for FSCAV data postanalysis. Hashemi’s group has also published work showcasing improvements to FSCAV’s temporal resolution, decreasing it from 1 m to 14 s.^114^

## Conclusion

More work is required to safely and effectively implement many of the monitoring techniques detailed in this review into clinical use. However, as far as clinical use goes, FSCV seems to stand out from the rest. Within the past decade, a plethora of studies have successfully demonstrated neurotransmitter monitoring with FSCV in humans. Lucio Boschen and colleagues provide an excellent review of the application of FSCV in humans.^118^ Unfortunately, recordings of serotonin with FSCV are outnumbered by the likes of dopamine. Nevertheless, even with the low number of successful recordings of serotonin in humans, given the biological importance of serotonin in widespread diseases like MDD and SUD as detailed in this review, it is worth briefly discussing these achievements.

In a study by Moran and colleagues, FSCV was used to record serotonin in the striatum of Parkinson’s disease patients undergoing scheduled DBS surgery.^119^ Their results showed that serotonin had a neuroprotective effect in patients engaging in high-risk behaviors manifested by increased serotonin concentrations. In a 2019 study, Montague and Kishida utilized novel computational methods that exploit FSCV features to allow for the concurrent detection of serotonin and dopamine in human subjects.^120^ Such an approach is a major advantage in studying a variety of neuropsychiatric conditions that involve the dynamic modulation and monitoring of multiple neurotransmitters at once.

These findings are extremely promising. However, in addition to the low volume of studies, the absence of studies utilizing absolute value measurement techniques is also notable. Thus, incorporating these techniques could be pivotal in changing the way we approach disease treatment in the future. For example, treatment for MDD and SUD requires long-term monitoring to examine their efficacy. MDD patients most often wait weeks to months after initiating treatment with SSRIs to know whether these medications will ultimately prove beneficial. Furthermore, MDD treatment commonly requires multiple office visits, during which the dose is constantly modified until a dose with optimal therapeutic effects is found. Nevertheless, even when optimal effects are attained, a plethora of physical and environmental factors can lead to a decrease in treatment success and thus require additional modifications to the regime. The development of a closed-loop system paired with a reliable neurotransmitter measurement technique could streamline this disease monitoring and treatment process. Such a system, ideally, would provide real-time monitoring of the disease via the detection of neurotransmitter concentration fluctuations, send this information to the healthcare provider or software, and allow for real-time adjustments to the treatment per clinical judgment. Though extensive work is still required to achieve this stage in medicine, advances in absolute value measurement techniques will likely provide the necessary tools to close the loop.

## Abbreviations

5-HT: serotonin
DA: dopamine
N-MCSWV: N-shaped multiple cyclic square wave voltammetry
FSCAV: fast-scan controlled-adsorption voltammetry
HPLC: high performance liquid chromatography
FSCV: fast-scan cyclic voltammetry
CFM: carbon fiber microelectrode
MDD: major depressive disorder
M-CSWV: multiple cyclic square wave voltammetry
DBS: deep brain stimulation
